# Why video health education messages should be considered for all dental waiting rooms

**DOI:** 10.1371/journal.pone.0219506

**Published:** 2019-07-16

**Authors:** Michelle McNab, Tony Skapetis

**Affiliations:** 1 Department of Education, Oral Health, Westmead Hospital, Western Sydney Local Health District, Sydney, New South Wales, Australia; 2 The University of Sydney School of Dentistry, Faculty of Medicine and Health, University of Sydney, Sydney, New South Wales, Australia; Hofstra University, UNITED STATES

## Abstract

**Objective:**

Video is an effective, accessible, and low cost method of delivering health education messages to a wide audience. Dental waiting rooms provide an opportunity to deliver video oral health education interventions to receptive viewers. In this study we aim to evaluate firstly video oral health education in regards to patient preference, and secondly its ability to change both immediate and sustained self-reported intended health behaviours by patients.

**Method:**

Data from 253 individuals from a public hospital dental waiting room were gathered using a previously validated survey following an oral health education video intervention, and analysed using descriptive analysis, Fischer’s Exact Test, and Wilcoxon Signed Ranks Test across 3 time intervals.

**Results:**

Participants across all ages evaluated the video oral health education approach as easy to follow and understand (p<0.001), the content practical and useful (p<0.001), and that it was a better experience (p<0.01). Those watching between 5 and 20 minutes reported that video was the best format to present oral care information (p<0.02). At follow up, significant improvement in the tools used by participants to clean teeth was seen (39.8%, p<0.001), as well as reported reduction in sweet consumption frequency (21.3%, p<0.001) and in smoking (44.8%, p<0.02).

**Conclusion:**

Video format oral health education used in dental waiting rooms was found to be effective in educating patients and instigating both immediate and sustained self-reported behaviour change. Significant improvement in tools used for oral hygiene and a reduction in sweets consumption were demonstrated, both of which are essential factors in reducing caries rates and improving oral health.

## Introduction

The provision of health education, and improved access to information, is aimed at improving understanding about available services, the causes of health and illness, and their personal responsibility for actions affecting their health. This enables individuals to make informed choices regarding the use of health care services as well as their own behaviour [1].

In the health care environment there is limited time available for patient health education, which is an essential component for the complete patient treatment and disease management [2]. There are a number of different media available to deliver health care information, including pamphlets, interactive multimedia, videos, posters and internet applications [3]. Health interventions and health education via DVD/video have been shown to provide a convenient, accessible and cost effective method to encourage a positive change and improvement in patient behaviour [3–5]. Improvement in plaque control and scores on post-test questionnaires have been seen with video instruction interventions in previous studies, indicating improvements in knowledge and conduct of oral hygiene procedures [6]. Studies have also shown greater patient satisfaction with education received during their clinic visit [2], and video health education interventions have been shown to be more effective than printed pamphlet information for both short-term behaviour change and long-term retention [7, 8]. Additionally, video format can also increase education uptake in individuals with low health literacy, and provide a standardised instruction and information presentation [3, 7]. Utilising DVD/video to deliver health care messages can also be more cost effective, less resource intensive, offers standardised messages, and can be delivered in a variety of forms, including DVD, video, USB, media file, and streaming video [3, 6]. The waiting room environment is an unavoidable component of health care, which can provide an opportunity to deliver health education messages to patients.

Previous available studies have shown that health education in video format in waiting areas has resulted in significant increases in health knowledge [9, 10] and allows health providers to focus on oral health treatment [9]. The presentation format that has been shown to be most effective consists of videos of real people taking a health related action [11]. Video health education interventions have also been used for a variety of people, including adults with special needs, youth and adolescents, and refugee and migrant communities [5, 12, 13].

There is a general lack of rigorous interventions that are of a higher quality and which result in health behaviour change [5] and a paucity associated with dental waiting rooms. Therefore, the aims in this study are firstly to evaluate if a multimedia/DVD format is preferred by patients when receiving dental health education, and secondly, its ability to change both immediate and sustained self-reported intended health behaviours by patients.

## Methods

The questionnaire instrument used in the study was developed using previously validated questions from four separate studies [14–17]. Questions evaluating DVD presentations were previously used in Chalmers et al, 2005, and Atkinson, 2007, and measure demographics, presentation qualities, aspects of the health information system, and the use of the DVD format [15, 18]. Questions used to evaluate change in dental knowledge were previously validated by Soutome et al, 2012, and Wagner et al, 2016, as well as those relating to oral health care behaviours [16, 17]. Additional questions used to identify aspects of behaviour change and DVD evaluation were tested prior to use through a pilot study. Free text comments were also invited.

The section of the questionnaire that evaluated oral health knowledge and behaviour was delivered in a retrospective pre-test style to measure both pre and post-viewing behaviour change. The retrospective pre-test method asks participants to give both pre and post-test responses after the intervention activity [19]. Participants rate themselves with a single frame of reference on both pre-test and retrospective post-test which helps to reduce response shift bias.

The oral health knowledge and behaviour section of the questionnaire was delivered to measure three time intervals, retrospective pre-test (T1), post-test (T2), and follow up (T3) conducted four weeks later. Questions were the same at each interval.

The questionnaire was piloted amongst Oral Health care professionals working in public health, including Dental Officers and Oral Hygiene Therapists, for content validity. Following minor changes, the questionnaires were further pilot tested amongst a small sample (n = 29) of patients from the target population and not included in the final cohort.

A DVD containing a number of Oral Health Education messages was presented in the main patient waiting area of Westmead Centre for Oral Health between March and June 2017. The waiting area used in this study services adult patients attending the Westmead Dental Hospital’s General Practice Clinic, Undergraduate General Practice Clinic, Acute Care Emergency Clinic, and Exodontia Clinics.

The messages in the DVD consisted of 12 video presentations, ranging in length from 30 seconds to 18 minutes, and approved for use by health authorities. The total length of the DVD was 1 hour and 9 minutes. The DVD included anti-smoking messages, oral care messages for adults and children, denture care messages, sugar reduction messages, and diet advice. The video segments were compiled from available health promotion resources developed by the Australian Government, the Centre for Population Health, the Australian Dental Association, the World Dental Federation, and the Centre for Oral Health Strategy. The format of the videos consisted of a mix of life performance and cartoon, and individuals featured included professionals from health services including oral health, community members and patients, actors, and media presenters. This array of video messages was utilised from a convenience sample of available, local health district approved sources. The video segments were arranged to ensure these messages were repeated often, to increase the exposure to the range of messages no matter when the participant began watching the presentation.

The study had full ethics approval from the Western Sydney Local Health District Human Research Ethics Committee (HREC) LNR/16/WMEAD/237. Written voluntary consent was obtained from randomly selected patients awaiting dental treatment while sitting in a large waiting room area. All participants involved in the study provided written informed consent and all participants were aged over 18 years.

It was anticipated that the response rate to the follow up survey may be low based on previous Australian surveys [20, 21] which reported 33.3% and 56% response rates respectively. A number of strategies were utilised to improve response rates including, frequent reminders, persuading respondents that their responses will be used, assuring anonymity of responses and keeping questionnaires brief as suggested by Nulty et al [21]. The 4 week follow up period at T3 is consistent with other similar published studies [22–24].

Statisticians were consulted to calculate power for the study with *n* calculated at 97 to achieve a statistical power of 90%. A sample size of 250 was chosen allowing for a 60% drop-off rate at T3 and is consistent with other similar published studies [2, 6, 7, 25–27].

Descriptive analysis was performed using SPSS Ver24.0 to calculate frequencies and the Fischer’s Exact Test was used in cross tabulations with significance set at *p* < 0.05. Additionally a Wilcoxon signed rank test was performed on responses from the pre- and post-test questions.

## Results

### Evaluation of the video health education approach

Pre-post (T1-T2) results as per Table 1, show that the majority of participants (62.8%) were aged over 45 years, were female (66.8%), and watched the video for less than 10 minutes (71.9%).

**Table 1:** Participant demographics (n = 253) at T1 & T2.

Variable	n	%
**Age**		
25 years or less	27	11.4
25–44 years	61	25.7
45–64 years	75	31.6
64 years or more	74	31.2
**Gender**		
Male	67	28.3
Female	169	66.8
**View time**		
Less than 5 minutes	60	28.6
5–10 minutes	91	43.3
10–20 minutes	52	24.8
More than 20 minutes	7	3.3

Agreement to each statement was defined as including a response of strongly agree, agree, and somewhat agree. There were high levels of agreement with all of the statements assessing the video content, as seen in Table 2.

**Table 2:** Evaluation of video presentation.

Statement	Agree (%)	Disagree (%)
The dental health video is better than using brochures or posters for learning	97.6	2.4
I found the video content to be practical and useful	96.8	3.2
The video was just the right length to watch	93	7
The video was easy to follow and understand	98.4	1.6
The video has prompted me to consider changing my own oral health care behaviour	90.7	9.3
I think other dental waiting rooms should have a dental health video like this one	96.8	3.2
I had more fun learning about my oral health because of watching the video	89	11
I learned about oral health more quickly and easily because of watching the video.	91.9	8.1
The video made learning about health a better experience than I would have had otherwise	95.1	4.9
Video was the best format to present this oral care information	95.9	4.1

Participants across all ages “found the video content to be practical and useful” (p<0.001), “easy to follow and understand” (p<0.001), and that “the video made learning about health a better experience than they would have had otherwise” (p<0.01). While there was agreement across all age groups who reported having “more fun learning about health because of watching the video”, this was seen to a higher degree amongst those aged above 45 years (94.5%) in comparison to those aged under 45 years (78.8%) (p<0.02). There was also a higher number of participants reporting having “more fun learning about health because of watching the video” who watched the video for between 5 minutes and 20 minutes (92.3%), in comparison to those who watched for less than 5 minutes (84.5%), and those that watched for more than 20 minutes (85.7%) (p<0.025). The majority of respondents reported that “video was the best format to present this oral care information” and was also significantly higher (p<0.02) among those who watched the video for between 5 minutes and 20 minutes (97.9%).

Comments from open-ended responses included positive feedback regarding the video content and presentation. Several participants stated that the volume of the video was too low, and that the messages were difficult to hear. There were also several comments made that the length of the video was too long, and some preferred the free-to-air television programming that was available previously.

### Follow-up survey results (at T3)

Response method, age, gender, and view time of the participants at the follow-up are presented in Table 3. The majority of participants responded to the follow-up survey by phone, were aged over 45 years (77.5%), were female (67%), and 41.7% of the group recalled having watched the video for 5–10 minutes.

**Table 3:** Participant demographics (n = 100) at T3.

Variable	n	%
**Method of response**		
By phone	79	79
Online	13	13
Mail	8	8
**Age**		
25 years or less	2	2.2
25–44 years	19	20.4
45–64 years	34	36.6
64 years or more	38	40.9
**Gender**		
Male	31	33
Female	63	67
**View time**		
Less than 5 minutes	24	28.6
5–10 minutes	35	41.7
10–20 minutes	22	26.2
More than 20 minutes	3	3.6

**Fig 1:**
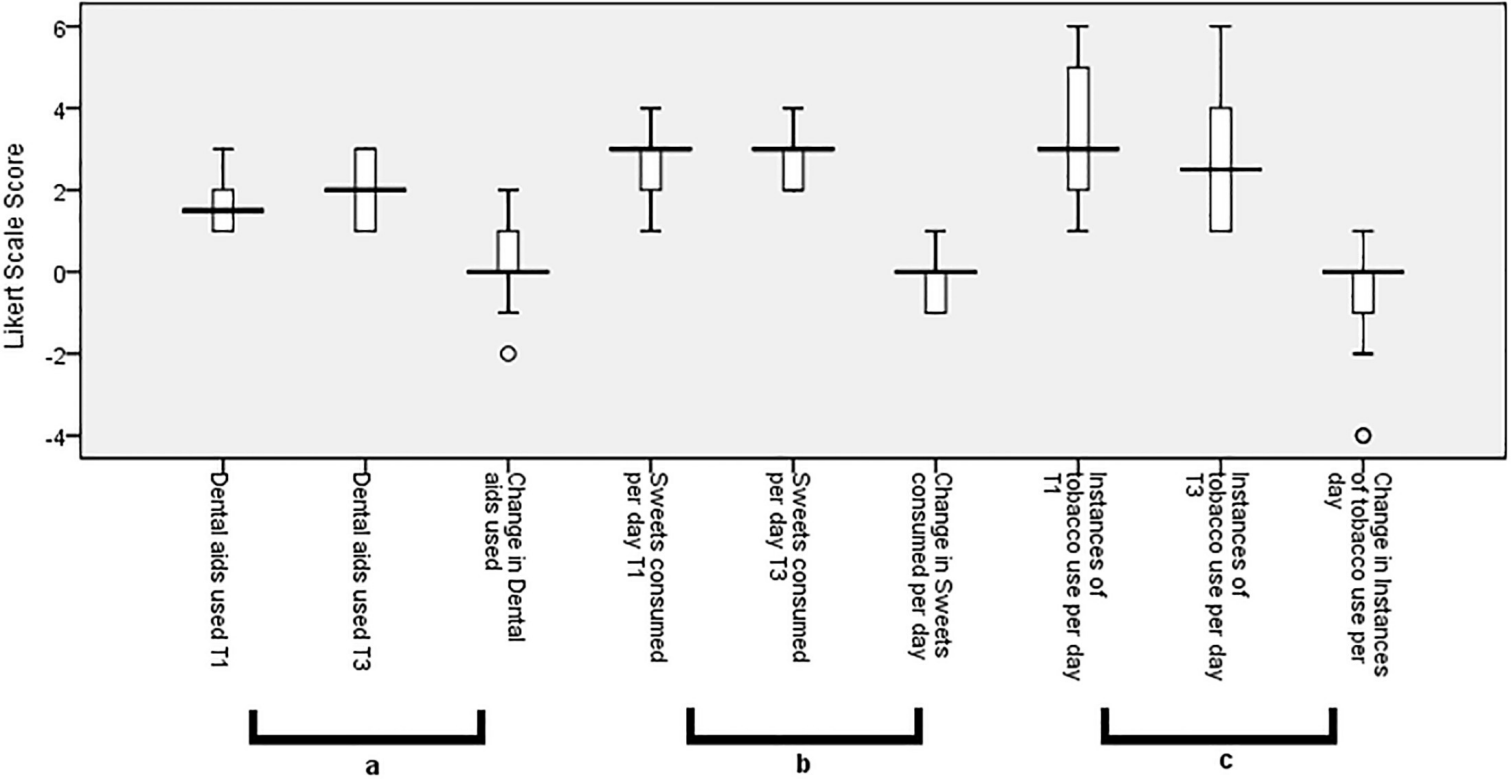
Significant change in self-reported behaviour between Pre-Test (T1) to Follow-Up (T3). a) Use of dental cleaning aids (tooth brush, interdental aids) reported at T1 and T3. Increase in the Likert score indicates increase in the use of dental cleaning aids. Significant increase in the use of dental cleaning aids was identified, b) Number of sweets consumed per day reported at T1 and T3. Decrease in the Likert score indicates a reduction in the number of times sweets were consumed per day. Significant decrease in the number of sweets consumed per day was identified. c) Instances of tobacco use reported at T1 and T3. Decrease in the Likert score indicates decrease in the number of instances of tobacco use per day. Significant decrease in the number of instances of tobacco use per day was identified.

Response changes from T1 to T3 can be seen in Fig 1. A significant positive change was found in self-reported behaviour through responses to the question “What kind of tools do you often use for cleaning your teeth?”. Over forty five percent (45.3%) of participants reported an intent to increase cleaning tools use between T1 and T2 (p< 0.001) and there was an increase in tools used by 39.8% between T1 and T3 (p<0.001).

Almost one third (32.8%) of participants reported intent to increase in number of times brushing per day at T1-T2, and for those who brushed less than 1 time per day, 79.2% reported intent to increase the number of times brushing per day (p<0.001). This intent to change was not carried through at T3, with no significant change in brushing frequency found between T1 and T3. There was no significant change to the number of times per day participants ate meals including snacks between T1 and T3.

One fifth of participants (21.3%) reported intent to reduce frequency of sweet eating immediately after viewing the video (p<0.001). This was also maintained at follow up, with 25.0% of participants reporting a reduction in sweets intake (p<0.025).

A large proportion of participants (80.9%) reported that they did not smoke prior to watching the video. Of those that did, 44.4% (n = 8) claimed an intention to reduce the number of times smoking per day after watching the video (p<0.01) and 44.4% (n = 8) reported a reduction in the number of times smoking per day at T3 (p<0.02).

Prior to watching the video (T1), 57.5% of participants were aware that tooth brushing should correctly start in infants with the eruption of the first tooth. This increased to 78.2% at T2 (p<0.001). At T3, this knowledge was not retained, with only 51.8% correctly identifying the age at which to begin brushing infants’ teeth.

The results showing significant changes in self-reported behaviour for the areas of the kinds of tools used for cleaning teeth, number of sweets consumed per day, and number of times smoking per day, were shown to be significant through Wilcoxon Signed Ranks Test and Chi-square tests. These significant changes in responses from T1 to T3 are demonstrated in Fig 1. Changes in tools for brushing were identified in the questionnaire as use of a toothbrush alone, use of a toothbrush and sometimes interdental cleaning aids, and use of a toothbrush and always interdental cleaning aids. Those that reported no smoking at all three time points were not included in the graph, to better illustrate the change in individuals that smoked.

## Discussion

This study assessed the response of patients in a waiting room environment to an oral health education video through self-reported behaviour and knowledge measurements. The analysis showed that the majority of the participants responded positively to the video, with high levels of agreement with the video evaluation statements, as shown in Table 2. Satisfaction with the video was expressed concerning the content, length, ease of understanding, and agreement that video was an ideal format to deliver health information [14].

Intent to change behaviour was indicated through agreement that the video prompted participants to consider changing their own oral health care behaviours. This was further reflected through the intent to change tooth brushing behaviour seen at T1-T2, with 32.8% of participants reporting intent to increase number of times brushing per day. Similar small but significant improvements in intent to change self-reported oral health practices through video format have been previously shown [7]. Video format may have advantages over other forms of health education resources, with 97.6% of participants agreeing that it was better than brochures and posters for learning. There was also agreement that learning about oral health through the video was more fun, allowed the learning to be done more quickly and easily, and was overall a better experience of learning, further supporting the relative advantage of video health education over other forms [14]. This has been similarly shown in previous studies indicating greater patient satisfaction with the education received during a clinic visit[2]. Furthermore, it has been reported that this style of oral health education video is effective in other similar waiting room settings [7, 15].

Over seventy one percent of the participants reported watching the video for less than 10 minutes with 41.7% watching between 5–10 minutes. Considering the short intervention duration, responses at T2 and T3 were similar thereby supporting the reliability of the survey instrument. This short viewing time is likely due to the nature of the waiting room environment, in which patients arrive shortly before their appointment time or are waiting in between appointments. None the less, positive responses to enjoying learning about health to a higher degree due to the video were seen more in participants aged above 45 years, and those who watched the video for between 5–20 minutes. This may be explained by the familiarity and popularity of television and video for those >45 years compared to younger adults who may be more engaged with their mobile devices [28]. One limitation of using this style of varied videos and lengths is the difficulty in ascertaining what messages were watched by each participant. In future studies, using a single short video might assist in ensuring participants are exposed to the same intervention.

Comments from participants stating that the volume of the television playing the video was too low, so that the messages were difficult to hear revealed a drawback to delivering this presentation in a waiting room environment. Additionally, almost one third of participants were beyond their mid-sixties which can indicate a higher rate of hearing compromise [29]. The volume of the video had to be kept at a moderate level so reception staff could easily communicate with patients. This meant that for some participants, particularly those who had hearing difficulties, the volume was not at an ideal level. In addition to this, when the waiting room was more crowded, there was an increase in background noise that could also make the video difficult to hear. Some participants suggested subtitles, although this might pose a problem for low literacy or culturally and linguistically diverse groups.

The majority of participants at T3 follow-up were female (67%), and aged over 45 years (77.5%), which may reflect visiting patterns showing higher numbers of females undertaking a dental visit within the past 12 months when compared to males [30]. Alternatively, this may reflect similar findings that females respond to survey administration at a higher rate than males [31].

The results show that there was almost 40% improvement in self-reported behaviour change surrounding use of dental cleaning aids at T3. Significant change in self-reported behaviour was also seen in the frequency of consumption of sweets per day, with 21.3% of participants showing intent to reduce frequency at T1-T2, and 25% reporting reduced frequency at T3 follow-up. This suggests that this type of video intervention may be effective in changing behaviour by reducing frequency of consumption of sweets, as well as improving knowledge and use of the different tools for cleaning teeth. This could be useful to deliver oral health instruction and diet instruction to patients while they are waiting for their dental appointment. A further explanation may be that whilst in a dental hospital waiting room, patients who invariably have a dental problem are more responsive to information about sweet consumption frequency and dental tool use and therefore more likely to attempt behaviour change.

The lack of significant self-reported behaviour change in terms of brushing frequency and meal and snack frequency could indicate that video information may not be effective in delivering these particular messages or that these habitual activities are difficult to influence. Changes in these behaviours may require more patient effort, longer timeframes, more individualised approach, or more frequent interventions to establish. Another explanation could be that people do not associate meals and snacks with dental problems unlike sweets.

Only a small proportion of the sample (n = 18) reported current smoking, 44.4% (n = 8) of which had intention to reduce their frequency of smoking per day. The same number (44.4%) reported a significant change in self-reported behaviour with a reduction in frequency of tobacco use at T3 follow-up. Due to the small number of smokers that took part in the survey this may not represent an accurate change due to the intervention. In addition, these results may be attributable to high numbers of current smokers (over 60%) that are considering quitting in the near future [32]. The nature of the survey also meant that the forms completed at T1-T2 were self-administered, and did not require any further interaction with research staff to answer questions, while at T3, those that responded by telephone had to provide their individual responses to the survey staff. This may have resulted in under-estimation of smoking, as smoking is viewed as a socially undesirable behaviour, which could be reflected as social desirability bias [33]. This may also account for the minor outlier for the change in smoking as seen in Fig 1.

Change in knowledge of oral health behaviours, while measurable at T1-T2, was not seen at T3 follow-up. This may have been due to the specific question measuring knowledge change relating to the oral health of babies, which may not have been considered relevant by the participants, especially as the majority were aged 45 and over. This was a limitation of the study, in which the survey was purposefully kept brief to encourage participation and completion by patients.

Other limitations of this study include response rate. The loss to follow up (60.5%) was not wholly unexpected and was consistent with similar studies [20, 21], but may have resulted in potential bias. To improve the response rate to this survey at T3, participants were contacted on several occasions, explained the importance of their participation, assured of anonymity, and the survey tool was kept brief [21]. Response rates may have been improved through face-to-face follow up interviews, or incentives [34], although this was not practical for this study.

The collection methods for T1-T2 were measured in person, while those for T3 were by phone, online or by mail. This is an additional limitation of the study and may have resulted in mode effect due to the differing collection methods, and resulted in social desirability bias. While this was considered during the development of the study, due to the nature of the public hospital clinic waiting area, which included patients attending for a single course of treatment with no planned follow up appointments, it was not possible to plan for follow up of patients in person.

A small number of questions measuring knowledge and behaviour were included in an effort to keep the survey short and manageable for participants and to improve response rates, but may not have provided a comprehensive indication of behaviour change. The use of multiple choice questions limits detail of the participants’ oral health knowledge and behaviour, and may have overlooked other learning outcomes.

There may have been an element of gender bias due to a large number of women participating in the survey and follow up, which may reflect higher participation rates by women in research surveys [31].

As far as we are aware, there has been no previous study examining the effect of a video oral health education intervention in a dental hospital waiting room, making this study a unique investigation into this environment, and enhancing the knowledge base for health education. To our knowledge, this is also the first time the retrospective pre/post-test style has been used in oral health education research. The study evaluated not only the satisfaction with video as a form of health education delivery, but also measured knowledge and self-reported behaviour change implied by the subjects over several time intervals with significant outcomes, enhancing its strength as a research study.

It might be useful to repeat this study looking specifically at the long-term effects of the intervention, and quantifying daily sweet consumption, possibly with the use of food diaries. This will provide a more detailed indication of changes in diet as a result of the intervention, and could be useful in directing the content of the video messages used. Additionally, further investigation is required to determine methods to overcome problems with hearing the health education messages. Further research in delivering the intervention to a larger group of smokers might also be valuable, to determine if there is a significant reduction in frequency.

## Conclusions

Video format is well received by patients and may be a preferable and more enjoyable method of oral health education. Furthermore, video is an effective tool to educate patients and to both instigate and sustain self-reported behaviour change. Video Oral Health Education messages should be considered for dental waiting rooms and were found to be effective in significantly altering and sustaining self-reported health behaviours in regards to frequency of sweets consumption and use of different oral care tools for maintaining oral health, both of which are essential elements in reducing oral disease and improving oral health. There may also be some effectiveness in the reduction of smoking.

## Supporting information

S1 Appendix Questionnaire. Previously validated waiting room questionnaire.
(PDF)
