# Supplementary material for: Why video health education messages should be considered for all dental waiting rooms
Source: PLoS One. 2019 Jul 16;14(7):e0219506. doi: 10.1371/journal.pone.0219506 (PMC6634391; doi:10.1371/journal.pone.0219506)
Supplement: S1 Appendix — Previously validated waiting room questionnaire. (PDF) [file pone.0219506.s001.pdf]

Waiting Room Video Survey

NSW GOVERNMENT | Health | Western Sydney Local Health District

We are evaluating our waiting room video and need your help. Please take a few minutes to fill out this survey and return it to the front desk. The information you provide will be used to help improve the content of the video and monitor the quality of this Oral Health Promotion Program.

Please tell us: Select (✓)

Your age in years:

❒ 25 years or less ❒ 25—44 years

❒ 45—64 years ❒ 64 years or more

Select (✓)

Your Gender:

❒ Male ❒ Female

Select (✓)

How long did you watch the presentation for?

❒ Less than 5min ❒ 5—10min

❒ 10—20min ❒ More than 20min

Please tick the box with the answer you feel best reflects your response.	Strongly Agree	Agree	Somewhat Agree	Somewhat Disagree	Disagree	Strongly Disagree
1. The dental health video is better than using brochures or posters for learning.	❒	❒	❒	❒	❒	❒
2. I found the video content to be practical and useful.	❒	❒	❒	❒	❒	❒
3. The video was just the right length to watch.	❒	❒	❒	❒	❒	❒
4. The video was easy to follow and understand.	❒	❒	❒	❒	❒	❒
5. The video has prompted me to consider changing my own oral health care behaviour.	❒	❒	❒	❒	❒	❒
6. I think other dental waiting rooms should have a dental health video like this one.	❒	❒	❒	❒	❒	❒
7. I had more fun learning about my oral health because of watching the video.	❒	❒	❒	❒	❒	❒
8. I learned about oral health more quickly and easily because of watching the video.	❒	❒	❒	❒	❒	❒
9. The video made learning about health a better experience than I would have had otherwise.	❒	❒	❒	❒	❒	❒
10. Video was the best format to present this oral care information.	❒	❒	❒	❒	❒	❒

Do you have any other comments regarding the video and the information provided?

Please tick ✓ the response that best indicates your answer with each statement BEFORE watching the video and AFTER watching the video.

BEFORE watching the video | **AFTER watching the video**

What kind of tools do you often use for cleaning your teeth?

❒	Mainly toothbrush
❒	Toothbrush and sometimes dental floss and/or interdental brushes.
❒	Toothbrush and always dental floss and/or interdental brush
❒	Toothbrush and tongue brush

After watching the video, what kind of tools will you use for cleaning your teeth?

❒	Mainly toothbrush
❒	Toothbrush and sometimes dental floss and/or interdental brushes.
❒	Toothbrush and always dental floss and/or interdental brush
❒	Toothbrush and tongue brush

Before

How many times do you brush a day?

❒	I don't brush every day
❒	1 time
❒	2 times
❒	3 times
❒	4 times

After

After watching the video, how many times will you brush a day?

❒	I don't brush every day
❒	1 time
❒	2 times
❒	3 times
❒	4 times

Before

How many times a day do you eat meals (including snacks between meals)?

❒	1 time
❒	2 times
❒	3 times
❒	4 times
❒	5 times
❒	6 times
❒	7 times or more

After

After watching the video, how many times a day will you eat meals (including snacks between meals)?

❒	1 time
❒	2 times
❒	3 times
❒	4 times
❒	5 times
❒	6 times
❒	7 times or more

Before

How often do you eat sweets?

❒	Many times
❒	Often
❒	Sometimes
❒	Not anytime

After

After watching the video, how often will you eat sweets?

❒	Many times
❒	Often
❒	Sometimes
❒	Not anytime

Before

How many ciga-rettes/cigars/ shisha do you smoke a day?

❒	None
❒	1—5 per day
❒	5—10 per day
❒	10—15 per day
❒	15-20 per day
❒	More than 20 per day

After

After watching the video, how many cigarettes/cigars/ shisha will you smoke a day?

❒	None
❒	1—5 per day
❒	5—10 per day
❒	10—15 per day
❒	15-20 per day
❒	More than 20 per day

Before

When should parents start brushing infants' teeth?

❒	With the 1st tooth
❒	1st birthday
❒	2nd birthday
❒	3rd birthday
❒	4th birthday
❒	Later

After

After watching the video, when should parents start brush-ing infants' teeth?

❒	With the 1st tooth
❒	1st birthday
❒	2nd birthday
❒	3rd birthday
❒	4th birthday
❒	Later

Thank you for your responses and comments, these are important to us. Please return this survey to the box at reception.
